# An Exploration of Psychosocial Pathways of Parks’ Effects on Health: A Qualitative Study

**DOI:** 10.3390/ijerph15081693

**Published:** 2018-08-08

**Authors:** Ewelina M. Swierad, Terry T. K. Huang

**Affiliations:** Center for Systems and Community Design, Graduate School of Public Health & Health Policy, City University of New York, New York, NY 10027, USA; terry.huang@sph.cuny.edu

**Keywords:** parks, green urban space, connection, health, wellbeing

## Abstract

Urban green space has been positively associated with psychological and physical health. However, the linkage between exposure to parks and health outcomes remains unclear. The current study examined the meanings that people assign to city parks, as a way to understand the pathways by which parks exert their effects on health. We conducted qualitative interviews with twenty culturally diverse residents in New York City. Thematic analysis was performed on the qualitative data. Results showed that all themes identified were related to parks fulfilling a basic human need for connection to (1) family, loved ones, and friends; (2) community and neighborhood; (3) self; and (4) nature. Based on these data, we proposed a human-centered framework for future research and interventions aimed at catalyzing parks as a vehicle to improve health and wellbeing. A human-centered approach emphasizes targeting the deep-seated needs and values of those we seek to engage and for whom health promotion and disease prevention efforts are designed. Our study shows that park transformations need to incorporate careful considerations of the human need for connection on multiple levels, so that park usage and its consequent health benefits may be optimized.

## 1. Introduction

Parks are lungs of the city—they bring life to urban space, allowing people opportunities for play, physical activity, recreation, social interactions, and personal and spiritual growth [1,2,3]. The evidence connecting urban nature and health is promising: city parks have been shown to be associated with increased physical activity [4,5], improved physical and mental health [6,7,8,9,10], lower body mass index (BMI) [11], reduced stress and anxiety [12], decreased morbidity [13], and even increased longevity [14]. Moreover, urban green space protects people from the detrimental effects of stress on their health [15,16] by decreasing heart rate, muscle tension, skin conductance, blood pressure, and inflammatory markers [17]. This buffering effect of nature on people’s health seems to be even more beneficial for underprivileged groups [18,19]. It is becoming increasingly clear that urban green space is an essential component of a sustainable city because it fuels psychological (e.g., supporting one’s own self-perception, sense of purpose, and stress reduction), social (e.g., strengthening relationships with others), and physical (e.g., allowing for physical activity beneficial for one’s physical and mental health) needs of its residents and it creates opportunities for a vast range of immersive and meaningful experiences [20,21].

Besides the health benefits that parks have to offer, access to green, clean, and safe parks is also a matter of social justice and equity [22]. According to previous research, as compared to White neighborhoods, minority (predominantly Black and Hispanic) neighborhoods were significantly less likely to have access to recreational facilities, and low-income neighborhoods were 4.5 times more likely to lack access to parks than high-income areas [23]. Moreover, research has shown that underrepresented groups such as African Americans, Hispanics, and foreign-born residents report more unique barriers to park use. These barriers include not feeling welcome and appreciated, cultural and language restrictions, inconvenient program schedules and pricing concerns, poorly maintained facilities, and safety concerns [24]. In the same vein, previous studies have found that limited spatial access to parks, inadequate or poorly maintained facilities, lack of bilingual staff, and perceived safety hazards were some of the biggest concerns regarding park use voiced by racial and ethnic minority groups [25,26,27]. Moreover, a recent study explored whether the quality of urban parks—measured through The Trust for Public Land’s Park Score—differs depending on a city’s median income and ethnic composition [28]. The researchers have found that affluent, majority-White cities have higher quality parks than those inhabited predominantly by low-income ethnic minority groups [28]. The study also found that cities with more residents representing a Latino group have higher neighborhood-level socioeconomic inequities in walking access to parks than cities with lower percentages of a Latino population [28]. Taken together, it is crucial to hear and integrate community voices, as ethnically and racially diverse as possible, into research and parks planning [18,22]. Yet, most research to date has focused on the influence of people’s mere exposure to parks rather than on the nuanced, individual meaning that they themselves assign to urban green space. Moreover, beside the salutogenic effects of parks on health [7,9,29,30], social interactions [3,15,31], and physical activity [4,5], the psychological mechanisms underlying these effects are not well understood.

Among plausible mediating pathways between city parks and individuals’ health and wellbeing is the sense of connection that people develop and negotiate with urban green space. Existing research examining psychosocial dimensions of parks’ usage has found that urban green space strengthens city residents’ relationship with other people, their community, and self [1,15,32,33], and improves their psychological wellbeing, including sense of purpose and connection to their community [2]. In the same vein, in their mixed-method study on psychosocial-spiritual benefits of engagement with New York City (NYC) parklands, Svendsen et al. [20] found that a variety of activities undertaken in urban green areas reflects a personal need to connect with nature and “a larger reality”, as well as with others and self. Utilizing interviews, field observation, and photo documentation, the study paints a picture of parkland as a space that extends beyond exercise and recreation and provides opportunities for human connection, self-reflection, and creative expression. Based on the results of their study, Svendsen et al. [20] suggested that parks are co-created and co-designed by nature and enable people “to feel part of the world around them, and perhaps to connect to a space inside them”.

Conceptualizing parks as a carousel of dynamic human emotions, relationships, and connection has important implications to urban planning, specifically human-centered design. This innovative, nonlinear, and multidisciplinary design strategy has recently gained popularity in the social and public sectors and has been used to address complex problems [34] including challenges facing public health [35,36]. According to the Hasso-Plattner Institute of Design at Stanford (d.school) [37], human centered-design teams follow a series of steps in the process of designing a particular solution, through: (1) Empathizing with the “customer”, (2) Defining (the problem), and (3) Ideating, (4) Prototyping, and (5) Testing possible solutions. The *Empathize* stage sets out to understand people, their way of doing things and the underlying motivation behind their actions, their physical and emotional needs, how they perceive the world around them, and what they find meaningful. The *Define* stage helps bring clarity and focus to the design process by articulating the design challenge accurately. The *Ideate* stage concentrates on idea generation and involves brainstorming concepts and outcomes that constitute the foundation for building prototypes and consequently innovative solutions to the design challenge. The *Prototype* stage consists of the iterative generation of potential solutions to the problem at hand and involves building “low-resolution, cheap and quick representations” of ideated solutions that can elicit useful feedback (in later stages of the design process the prototypes may become more refined). The *Test* stage focuses on soliciting feedback about the prototypes, gaining empathy for a target audience in the context of the designed solution, and refining design solutions further. As these design stages exemplify, at the core of the human-centered design methodology is a fundamental belief that in order to design innovative and effective solutions, it is essential to understand people’s needs, values, perceptions, and behaviors and to engage with them at every level of the design process [34]. Designing human-friendly parks is not an exception.

In this context, the current research examined individuals’ perceptions and personal meanings of parks as a way to hear the voices of communities and to provide ideas for an implementation of their identified personal meanings into the design of parks. The study built on previous research that explored psychosocial dimensions of urban green spaces [15,20,32,33], but it is distinct in the following ways. First, the previous studies integrated a variety of methods simultaneously (e.g., observations, photography, interviews) in exploring psycho-socio-spiritual aspects of park use—in contrast, the current study exclusively focused on in-depth analysis of self-reports as a way to unravel the unique psychology of park use. As such, the nature of the research was more psychological and intrinsic in scope as opposed to focused on social dynamics or spiritual meanings of a physical space alone. Concentrating exclusively on the psychology behind park use, the researchers explored individuals’ perceptions of urban parks in order to identify psychological mechanisms by which parks exert their effects on health. Specifically, personal meaning of and relationship with parks were examined. Second, the study was contextualized within and inspired by the principles of human-centered design, and the outcomes and implications of the study were explored in the context of a human-centered design framework. Third, it utilized in-person interviews with culturally diverse New Yorkers representing predominantly underprivileged communities, as opposed to with just one sociocultural or particular user group [32,33]. Fourth, in contrast to a structured set of questionnaires [32,33] or rapid interviews [20], the study deployed open-ended questions that exclusively focused on participants’ personal reflections on city parks and that allowed the researcher to develop a rapport with research participants. The opportunity to develop trust with culturally diverse individuals afforded by conducting in depth qualitative interviews is often cited as an essential ingredient for ethical, informative, and culturally sound research [38]. Given that trust creates a safe space for people to open up and reveal their genuine thoughts and feelings, qualitative interviews help to develop implementation strategies that reflect people’s deeply seeded needs, values, and beliefs [38]. Finally, this qualitative research is a sub-study of a larger research project—the PARCS (Physical Activity and Redesigned Community Spaces) Study. The PARCS study leverages a natural experiment opportunity to examine the impact of the Community Parks Initiative (CPI)—a citywide redesign and renovation effort in New York City—on physical activity, park usage, psychosocial and mental health, and community wellbeing [39].

In the current qualitative PARCS sub-study, twenty PARCS study participants were asked the following questions: (1) What does green urban space mean to you? (2) What comes to mind when you think of parks and green urban areas in your neighborhood? (3) What does participation in the PARCS Study mean to you?

Following the review of the themes identified from the interviews, the results are discussed in the context of urban planning and human-centered design. We argue that a human-centered framework could inform future research and interventions aimed at using parks as a vehicle to improve health.

## 2. Materials and Methods

### 2.1. Research Design

This PARCS sub-study used an open-ended interview format to examine the perceptions of and personal meaning that people assign to parks and urban green areas. The interview format enables researchers to obtain detailed insight about participants’ experiences, perceptions, beliefs, values, and behaviors [40], which can be particularly important in examining concepts that are relatively unexplored, such as personal meanings of urban green space.

### 2.2. Participants and Recruitment Strategies

Participants were 20 ethnically diverse individuals selected from among PARCS study participants living in low-income neighborhoods in New York City. They included participants from two groups that varied on the level of adherence to the PARCS study protocol (i.e., they were pulled from a group of 50 that were high-adherence and 50 that were low-adherence individuals). These individuals were randomly selected by a researcher not involved in the current qualitative study. The lead author of the current paper, who is otherwise not involved in the recruitment or data collection of the larger PARCS study, then contacted individuals on the list by phone to invite them to participate in the interview.

Out of 20 participants who agreed to participate in the interview, the majority (i.e., 70%) were highly adherent PARCS study participants. There were no significant demographic or socio-economic differences between high versus low adherent groups. Participants were selected from across 54 park neighborhoods that are part of PARCS. PARCS targets predominantly residents from public housing (New York City Housing Authority or NYCHA). PARCS also recruits non-NYCHA residents if they indicate that they have lived in the neighborhood for at least two years, intend to stay in the neighborhood over the next four years, or are otherwise engaged in a community organization. Participants of the PARCS study live within the 0.3-mile buffer of each study neighborhood. The PARCS Study includes adults ≥18 years of age with no mobility issues and who understand/speak English, Spanish, or Chinese (Mandarin or Cantonese) [39]. Out of twenty participants of the current qualitative study, 17 were women, 3 were men; 5 self-identified as Hispanic or Latino/a, 6 as Black or African American, 5 as White or Caucasian, 4 as Other. Just as with the larger pool of PARCS study participants, some individuals invited to the current qualitative study were NYCHA residents, and some were non-NYCHA residents. Eight participants reported completing high school or below; another 8 reported completing either some college or technical school or graduated from college; and 4 participants completed graduate or professional degrees. Seven participants were severely low income, with household income of less than $20,000.

### 2.3. Procedure

Commencing each brief interview, the researcher introduced herself and explained the purpose of the study. After providing informed consent, the researcher read the interview questions in sequence. This format involved using three open questions that explored participants’ perception of urban green space.

Interviews were held over a 4-month period and the researcher met with the participants at their local cafés. The brief interviews aimed to explore three research questions: (1) what is the personal meaning of parks in people’s lives? (2) how do people perceive their personal relationship with urban green areas? and (3) what are the mechanisms behind the beneficial influence of parks on individuals’ health and wellbeing? Interview questions were inspired by and developed based on literature review of studies related to psychosocial-spiritual dimensions of parks that identified increased psychological and spiritual wellbeing as a result of spending time in green spaces that are perceived as “beautiful” and “relaxing” and that provide opportunities for cultural activities and nature recreation [32,33] as well as for connection and self-expression [20].

These questions were explored as part of a broader interview of PARCS participants that lasted about one hour per person. The interview questions were piloted with three PARCS study participants.

### 2.4. Thematic Analysis

The interviews were transcribed and de-identified. Each participant was assigned a number (e.g., “P1”). Interview transcripts were then imported into NVivo version 10, a qualitative data analysis computer software package. Thematic analysis [41] was used to identify common themes and patterns related to participants’ meaning that they attach to their parks. This qualitative method recognizes language as a tool for storytelling that reflects people’s broader social and physical environments [41]. In other words, thematic analysis highlights the ways in which people make sense of their experiences that are informed by their social and environmental context [41]. Understanding the context of people’s experiences seems to be particularly important in the case of members of underprivileged groups (who represent the majority of PARCS study participants) that are often discriminated against and overpowered by external, systemic forces affecting their lives [42]. Therefore, this method, as a supplement to the larger quantitative PARCS study, is well suited to examine the perceptions and experiences of the study participants.

The interview data were analyzed following the thematic analysis guidelines created by Braun and Clarke [41]. During each stage of the analysis, the authors of this manuscript discussed the emerging themes to ensure the consistency within patterns identified.

## 3. Results

Table 1 presents four broad themes that were identified throughout the interviews. All themes are related to parks fulfilling a basic human desire for connection to (1) family, loved ones, and friends; (2) community and neighborhood; (3) self; and (4) nature.

### 3.1. Theme 1: Connection to One’s Family, Loved Ones, and Friends

This theme reflected the common belief that city parks provide participants with the opportunities to connect with their family members, loved ones, and friends. The majority of participants mentioned that city parks allow them to spend time and strengthen ties with their loved ones engaging in a broad range of activities. For example, *P6* discussed the importance of parks for nurturing her relationship with her son as follows.

*P6:* “Me and my son go to parks to forget about the stress of life, to enjoy time together, even if it is just half an hour (…). My son and I use parks quite often—for us it is about family time, we bring a dog sometimes, or a soccer ball, or sometimes we just go for a walk, depending on our mood or weather”.

Similarly, *P3* described how parks are an essential space for enjoying family time:

*P3:* “I love parks, and I take my kids to the park a lot. We go to the park every day after they finish school. They bike there, or play basketball when the weather is nice. I also meet there with my friend, and we chat while kids are playing. We use parks constantly and we are excited to see how the park will change after the renovation”.

In the same vein, *P8* noted:

*P8:* “I go to my local parks with my family, we play together, we have a good time; sometimes our friends join us (…) we play basketball, volleyball, it is great. In the summer we go to parks a lot; we can spend even an entire day in the park”.

Other participant (*P10*) argued that while parks can mean different things to him depending on the life stage that he is in, right now green urban areas are about spending quality time with his daughter:

*P10:* “Parks are like baseball, they mean different things to you in different points of your life—right now it is a place for my kid where she rides a bike, so right now for me parks mean play and time spent with my daughter (…) the first six years of her life—we went to park almost every day (…) also she plays hockey in Central Park once a while now—so parks are very important for us for that reason too”.

A number of participants identified the idea of ensuring that they do whatever it takes to make parks nicer and greener for their own children in the future and for the next generation. For instance, *P1* noted:

*P1:* “I have grandkids, and I want to leave something for them clean and safe environment. What is important to me now is happiness and health and it often has to do with the environment, it is about leaving this place better and greener for the next generations (…) Health is essential in life and you have to have good schools, housing, and clean environment to get it. You cannot have a good health if you do not have a good environment”.

Similarly, *P4* highlighted the importance of safe and green city parks to the next generations:

*P4:* “In my park they show movies once a while; they have tables where kids can sit and eat after they play and it is a safe environment for the kids and it is great for the community to have a place like that (…) everybody cares about what is going to happen to your loved ones, your children after you are gone, so parks are about making things better for the next generations”.

A few participants, such as *P5*, suggested that spending time at parks is an opportunity to explore one’s neighborhood and teach children about the community that they are part of:

*P5:* “My children love parks. I want to show my kids everything, I want to teach them, let them experience life. I want my children to be part of something that is meaningful, purposeful. Through our participation in the PARCS study we have been learning about our neighborhood (…) we walk places and we learn. One time we had “*a reading a book event*” in the park—it was a great exercise, it was like being in a library, but outside”.

In addition to teaching children about their communities, some participants argued that spending time with family at parks can be a great opportunity to teach children about the importance of clean and green environment. For example, *P17* remarked:

*P17*: “You can make children appreciate parks by explaining to them what we get from green spaces, parks, like fresh air or interaction with family (…). The first thing that comes to mind when I think of parks is relax with my family”.

### 3.2. Theme 2: Connection to One’s Community and Neighborhood

This theme expresses the notion that through engagement in parks, people feel connected to their local communities and broader physical neighborhood. Many participants identified the importance of cultivating their relationship with their immediate social and physical microcosms. For example, *P18* commented:

*P18*: “Parks bring people in a neighborhood closer together. Because if you sit in a park, you are going to talk to somebody, you need to meet new people, know your neighbors that you did not even know are your neighbors”.

Similarly, *P2* noted:

*P2:* “Sometimes, where we are on a playground and my son is busy playing, I talk to other parents—that’s my chance to talk about different things with them while kids are playing”.

Some participants discussed how parks are great places for connecting with people from the community who share similar interests and who like to engage in the same type of activities. For instance, *P2* reported:

*P2*: “I am a recreational runner—I join the group of people from my community every Saturday in my park in Astoria and we run for three miles—it is great to run with other people, to have company (…). There are different groups of people in the park—and it is very interesting to me to watch people. I have seen runners sticking together, as well as board skaters, or even vintage car collectors driving around the park to show off. Recently I saw a person flying a drone—and he let me use the remote so I experienced it myself, controlling a drone and we took unbelievable pictures of New York, above the bridge, above East River, and it all happened because I met a stranger in the park and had a conversation”.

Parks seem to be the places that bring people together allowing them to immerse themselves in new and enriching experiences. The comment of *P2* provides an illustration of how parks attract a broad range of artsy and creative initiatives that enable people in a community to participate in activities that they would have not otherwise had a chance to partake in.

*P2:* “In Astoria they organize concerts and movie shows at my park—even symphony orchestra comes once in a while in the summer, imagine, in the park!!! I never have seen symphony orchestra outdoors before—that was different, but great experience. It was amazing for everyone in the neighborhood—not everybody can go to Carnegie Hall or Lincoln Center. I learn about different activities organized in my park—they have brochures with all the scheduled activities, concerts, movies, exhibits of modern art; and they also have boards with all the activities scheduled for the month; last year they had movies from different cultures—every single Wednesday; in one of the parks in Queens we have sculptures—I love going there, sitting on a bench, and eat a meal, overlooking Manhattan (…) And one of my favorite activities in the summer in Astoria Park is the pool, more than 2000 people capacity—it is very nice; many families come as the pool has different levels—so you see babies, adults swimming and enjoying the pool”.

Many of the participants discussed their participation in the PARCS study in the context of connecting with their communities and making a difference. They highlighted the importance of engaging in initiatives that contribute to a cleaner and greener environment as well as healthier and happier communities. For instance, *P17* remarked:

*P17* “I participate in PARCS study because I want to see our community better; our society kinder, working together to make a difference in our lives and in the lives of our children”.

Similarly, other participants shared a belief that engaging in activities focused on improving the quality of people’s communities and neighborhoods is their civic duty and one of the ways to be an active co-creator of their city landscape. Here is what *P10* stated regarding this point:

*P10:* “You cannot complain about New York and not participate in a study (PARCS) like this that makes our city better; and if there is a data that shows that—and I hope your data will show that—it is going to make the city more motivated to do more renovations, and consequently improve lives”.

In the same vein, another participant (*P4*) mentioned that only those who participate in different initiatives that make their community greener and healthier are allowed to complain if they do not like the direction in which things are going. *P4* articulated this belief in the following statement:

*P4*: “I’m part of PARCS study because I like to have a voice when it comes to my community, my neighborhood. Otherwise, I’m not allowed to complain when things go wrong. My husband and I are all about making things better for the community”.

Another participant (*P13*) expressed the same belief:

*P13:* “Parks are about the future of our communities and neighborhoods. I feel like by participating in this study I’m making a difference for my city, I’m shaping the future of my city”.

The majority of participants mentioned that having and expressing their voice about the future of New York and its parks and green space was essential for them and reflected their attachment to and connection with their communities. They spoke about the fact that seeing the renovations and redesign of their local parks made them feel good and brought a sense of hope for the future of the city. For example, *P20* remarked:

*P20*: “So first I want to mention that I work close to the park that has been renovated, and when I saw them fixing up the park—which made me feel great to see that change in my community—I thought that by participating in the study I have something to do with it, I have a voice (…) I always try to do nice things for my community”.

Similarly, other participants shared their excitement about the renovation of their local parks and reflected on their ideas about ways in which parks could bring value to their communities. For instance, *P17* noted:

*P17:* “I’m so glad to see the park being renovated in my neighborhood, because it is good for all of us, for our community. I would like to see more group activities organized in my park for the community to enjoy, like soccer game, concerts etc.”

Another participant (*P4*) gave her suggestions as well:

*P4:* “I like the fact that through your study my voice will be heard, that I have a voice about my community so that my park stay modern and clean (…) I would like to see different events at parks that are good for the community, something fun for everyone, no matter how old they are, for instance a book swap—people come in and bring books that they have read already and they trade them for a new books or magazines”.

These findings indicate that people tend to think about parks in the context of their connection to their community and local neighborhood. They perceive city parks as spaces that allow for different experiences aimed at connecting to one another. In addition, having a voice about the future of people’s urban green space and making a difference in their environment appear to be important to PARCS study participants.

### 3.3. Theme 3: Connection to Self

In addition to seeing green space through the lenses of connection to family, loved ones, and their communities and neighborhoods, many participants consider parks to be a safe space for reflective self-exploration that facilitates personal growth. For example, discussing the importance of parks in her life, *P2* stated:

*P2:* “I would say to anyone, go out there, get fresh air, run, walk, it is good for you, it is healthy—it is good for your health, your emotions, your body, soul, and mind (…). Parks are great places for relaxation; place where you can leave all the problems behind, connect with yourself, immerse yourself in complete rest and relaxation”.

Some participants argued that parks are essential for accelerating their recovery from different health conditions. They provide them with a sense of healing, space that speeds up the recovery process. For instance, *P9* reported:

*P9*: “Parks help people to stay healthy; now recovering from a serious condition, I spend a lot of time in parks, it helps me—you’ve got a bench, you sit down, you wander around the park, it is a good place to be at, to study—it is good to be out and about and have some fresh air. I use parks a lot”.

Similarly, *P10* remarked:

*P10:* “I hope your study will support the notion that being close to a park is good for your physical and mental health—because it is”.

Many participants perceived parks as areas in which they can recharge their energy, enhance their “physical and mental health” (*P19*). They discussed the need to connect with themselves in the context of disconnecting from the hustle and bustle of their busy city lives. Many of them perceived parks as space that helps in alleviating daily life stressors, just as *P11* pointed out:

*P11:* “For me being in a park is relaxing; it is about disconnecting from stressful life and breathing a fresh air, walking around, reading a book, playing with kids, just relaxing, enjoying the sunshine and forgetting about the city.”

Emphasizing the importance of parks in improving one’s wellbeing, *P19* noted:

*P19:* “It is important to me to get out of the house and exercise in a park, to get fresh air, I like when parks are colorful, playful, it makes you feel good”.

Some participants discussed how parks allow them to spend time on their own, alone, “to watch people, have a conversation with a stranger” (*P2*) or to be immersed in in their favorite activities that bring them joy. For instance, *P17* commented:

*P17:* “I love taking pictures in the park, it relaxes me. I take pictures of everything, a flower, a dog, a bird, a tree, the sun, the sky, the moon (…). It makes me thankful to be alive, appreciative.”

Similarly, *P2* noted:

*P2:* “While the kids play, I sit down and enjoy my time, I look at other people, sometimes, I talk to other parents, or sometimes I go to the park alone—I take my dog for a walk and I enjoy that too”.

### 3.4. Theme 4. Connection to Nature

The final key theme identified from the qualitative findings was participants’ connection to nature as a driving force for their local parks’ visitation. Many people expressed the sentiment that parks “give us fresh air and contact with nature” (*P20*); people perceive green urban space as the oasis of peace that allows them escape from the noisy concrete jungle. For instance, *P16* remarked:

*P16:* “This life is very hectic so we need to remind ourselves to come back to green world, parks, and green areas in the city. Whenever I have time, I take my kids to the park and we are active”.

Similarly, another participant (*P2*) underscored the importance of nature in people’s relationships with parks. She mentioned:

*P2:* “I think it is very important that more people go to parks to be close to nature—I see in the summer people bringing blankets, having small picnics, and enjoying the greenery around them”.

In the same vein, discussing the reasons for visiting parks, one of the participants (*P5*) described the richness of natural life that parks have to offer:

*P5:* “I love the parks because I love the trees, animals, birds etc.”

Many participants discussed how the greenery of parks improves their mood and wellbeing. For instance, one participant (*P17*) stated:

*P17:* “I love sitting in parks with the kids—they play and you look around, watch them, and the greenery keeps you calm; now we see a lot of buildings raising, but parks are more important to us”.

Some participants anticipated that contact with nature will become a more essential drive for visiting parks once they get older. For example, *P10* noted:

*P10:* “Right now for me parks mean play and time spent with my daughter, but later, when I’m 80 I’m sure it is going to be a place where I go and relax and spend time surrounded by nature, in a green space”.

These findings suggest that nature and its calming and soothing effects on people’s health and wellbeing remains one of the most important factors that facilitates their engagement with city parks.

### 3.5. Summary and Implications—A Human-Centered Framework to Understand the Potential Implications Regarding the Effects of Parks on Health and Wellbeing

**Figure 1:**
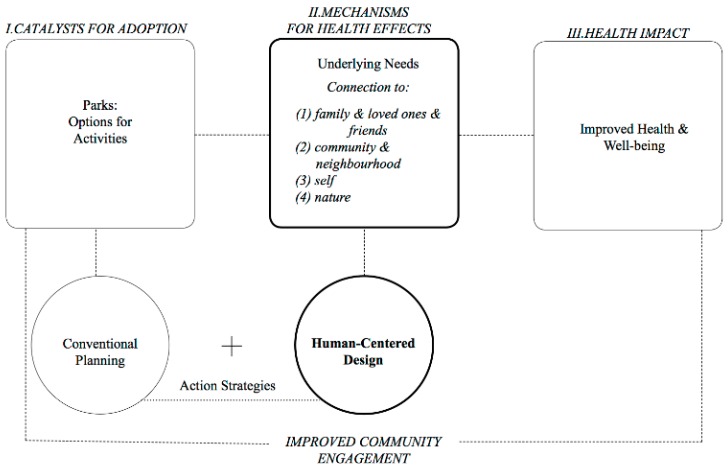
A human-centered framework to understand potential implications regarding the effects of parks on health and wellbeing.

Figure 1 presents our proposed human-centered framework that may be helpful for future research to explore and to understand the psychosocial pathways via which parks may exert their effects on health and wellbeing. Taken together, one primary implication of our results is the importance of expanding the conventional urban planning strategies and incorporating psychosocial factors into the design of urban green space. Given its focus on human perception and experience, human-centered framework can provide one of the ways in which the design of parks can incorporate essential human values, needs, and beliefs. We propose that at the core of this framework is the idea that parks need to validate the human need for connection. Specifically, the results of this study identify various levels of connection as potential mechanisms that might underlie individuals’ engagement with their green environment in the city. The need for connection seems to drive people’s engagement with parks and expresses itself in individuals’ relationship to (1) their family and loved ones; (2) their communities and neighborhoods; (3) their inner selves; and (4) nature. Given the importance of connection in our lives and their beneficial effects on people’s health and wellbeing [43,44,45], it seems natural that the human desire to connect governs our relationship with urban space as well. A human-centered design approach can help urban planning and public health professionals better explore individuals’ deep-seated needs and values [46], which has the potential to improve community engagement [47] and consequently enhance people’s health and wellbeing.

The current study explored the center piece of this framework (bolded box) showing the potential psychosocial pathways that connect parks to health outcomes. This hypothetical model proposes that conventional planning may benefit from integrating into its structure a human-centered design to enhance park usage and community engagement and improve health. It should be noted, however, that exploring causal relationships between the concepts and ideas depicted on the graph was beyond the scope of this research. Despite this limitation, the framework captures major concepts that emerged in the study and may serve as a basis for further investigation of causal links between these concepts.

## 4. Discussion

The results of this exploratory study illustrate the idea that urban parks are not only the lungs of the city, providing people with opportunities for physical activity and improving their health and wellbeing, parks are also the heart and soul of the city, enabling people to develop a genuine and emotional connection to the world around them and to themselves. Indeed, consistent with previous research [20,32,33], the current study found that using green urban space is, in part, motivated by humans’ deep desire to connect with their loved ones, but also with a larger community around them, with nature, and with their inner selves. This finding is aligned with decades of psychological research that has identified connection as one of the most fundamental human needs that allows people to flourish and feel seen, heard, and valued for who they are [21,48,49,50,51]. Our brains are hardwired to connect with one another and with the broader world around us [52]. These connections with loved ones, friends, and communities are just as important to our survival and flourishing as the basic needs of food, safety, and shelter [49,52]. In fact, in his hierarchy of needs, Maslow places social connection just after basic physiological and safety needs [50] and identifies self-actualization—a deep connection with self, expressed in our quest for self-knowledge, meaning of life, or beauty—as one of the basic human needs as well.

Because having a sense of connection with others, our communities, and our inner selves improves our mental and physical health [43], it is essential to promote and design spaces that facilitate all forms of connection to boost the health of our communities. According to previous research, various forms of connectedness increase our capacity to tackle and overcome adversity and to improve our health and well-being more generally [45,53]. In fact, because of their beneficial effect on health and wellbeing, connectedness and sense of belonging to different groups have recently been described as “social cures” [44,45]. Researchers have started exploring ways to make connectedness policy relevant to improve health outcomes for specific populations and society in general. They explore the question of how policy can facilitate the development of sustainable and socially engaging communities to promote physical and mental health [43]. This understanding can be applied to urban green space as well. By creating opportunities for connectedness and designing policies that promote all forms of connection discussed in this paper, we can collectively benefit the health of our communities, especially disadvantaged individuals whose health is often jeopardized.

Promoting all forms of connection through green urban space is more important than ever given current social, cultural, and demographic trends emerging across large metropolitan areas in the United States (US). For instance, because more than 50 percent of American adults are single, and 31 million—around one out of every seven adults—live alone [54], connecting with others and our own needs seems to be important to our wellbeing and sense of connectedness. Moreover, a typical American family size has become smaller over the years [55], the rate of divorce has been increasing [55], and people are spending more time in the virtual world, disconnected from their loved ones in the real world [56]. These socio-cultural trends that have emerged within the social fabric of our lives call for revisiting and examining more closely our need for connection. In addition, in a multicultural country like the US, immigration has remained a factor that enriches the social and cultural landscape of our communities, creating opportunities for connecting with others from different backgrounds. Through proximity with ethnically diverse individuals and communities, we broaden our horizons and worldviews and become more educated and culturally aware citizens. Connecting with others who are different than us has become even more critical in times where the country is more polarized than ever because of the current socio-political climate that does not promote inclusion and appreciation of diversity.

Along with previous research highlighting the importance of individuals’ social, psychological, and spiritual dimensions of park usage [1,20,32,33], the current study emphasizes the human need for connection as a driving force of people’s engagement with their parks. These results are also aligned with the outcomes of the Design Thinking for Parks workshop that our group has been experimenting with to gain insights into the needs and values of the PARCS study participants and to co-create ideas for park engagement [57]. Corroborating the results of the current study, our workshop has revealed that tools to enhance the connectivity of residents could potentially enhance their park use. Participants of the workshop described parks as an extension of home and as safe spaces that are physically and emotionally accessible to everyone where people engage in activities that foster self-improvement and personal growth. As part of the community participatory ideation process, some design prototypes that may facilitate connection to people’s family and loved ones, to communities and neighborhoods, to their inner selves, and to nature included: (1) an interactive screen/web space that would help inform individuals about events happening at their local parks—if people know about the events organized at their local parks, they may be more likely to attend them, and consequently connect with one another, with nature, and depending on the character of the event (e.g., educational, inspirational), with themselves; (2) designated park areas for vendors to do business, created for local businesses to gain community exposure and to benefit that community—this would allow urban residents to connect to their neighborhoods and communities by supporting a variety of local businesses; this initiative would also provide an opportunity for the exchange of ideas between different business and service providers and customers; (3) a digital storytelling platform with a physical screen in parks for community members to share their stories, talents, culture, etc. In addition, there could also be a small amphitheater with a central focus area, similar to a stage, that would afford urban residents storytelling opportunities. The platform can facilitate connection between like-minded individuals, expose others to a variety of artistically and aesthetically enriching performances, and allow people self-expression and self-exploration, enabling them to connect to their inner selves as well.

Although the current study revealed some important insights regarding the personal meaning that individuals assign to their parks, there are some limitations that should be acknowledged. First, our sample was focused on residents in low-income neighborhoods in NYC. Therefore, study findings may not be generalizable to all populations. Future studies can build on the insight derived from this research and measure different dimensions of connection in a quantitative manner with a larger and more diverse sample of participants. Although questions designed for the study were carefully crafted and tested to ensure cultural sensitivity and accuracy, our question set was limited, and a different set of questions could have yielded different results. We included only open-ended questions, which in qualitative research are commonly used as a basis for theory development, exploring relatively unknown research areas [58,59] such as the personal meaning and perception of urban parks. This format addressed the research goals and allowed for free exploration of participants’ views. Finally, the sample consisted predominantly of women—out of twenty participants, only three were men. This disproportional rate of women to men could influence the results of the study as women may perceive connection to family and friends and their neighborhoods and communities as more important than men typically would. The importance of human connection in women’s lives can be a result of a typical socialization processes that still highlight nurturing the relationships with others as more important for women than men. Future studies should explore how men, as compared to women, perceive the notion of parks as space that facilitate interpersonal connection.

## 5. Conclusions

Urban parks are not only the lungs of the city enabling people to breath fresh air and stay healthy; parks are also the heart and soul of the city—they beat in the rhythm of our own hearts letting us develop emotional connections to the social, physical, and spiritual world around us. Previous research [1,20,32,33], the current study, and the lessons from our experimentation of human-centered design suggest that parks are the places where people from all walks of life express themselves, look for connection, and search for what they need and value. As such, parks are like canvases, allowing people to paint different images that symbolize various meanings and convey personal stories. Usually these stories are about connection. Consistent with previous evidence [60], our study suggests that park improvements may not necessarily and automatically increase park usage—park transformations may need to incorporate careful considerations of the human need for connection on multiple levels, so that park usage and its consequent health benefits could be optimized. In a technology-driven world that often disconnects us from one another, revitalizing the basic human need for connection through urban parks seems to be more important than ever. Providing opportunities for connection is critical in large urban environments like New York City where people live so close to one another, yet in the hustle and bustle of their busy lives, they may feel lonely and isolated all the same. Therefore, urban green space may serve a dual purpose of providing quietude, letting us connect with our inner selves, but also opportunities for social connections with others to strengthen the formation of community and a sense of belongingness. Perhaps green urban spaces, among other things, led Tom Wolfe to say famously that “one belongs to New York instantly, one belongs to it as much in five minutes as in five years”, because New York City and its parks are an “extension of our homes”.

## Figures and Tables

**Table 1:** Themes identified from the qualitative study.

Theme	Brief Description
1. Connection to one’s family, loved ones, and friends (e.g., “*Me and my son go to parks to forget about the stress of life, to enjoy time together, even if it is just half an hour (…). My son and I use parks quite often—for us it is about family time*”)	City parks provide participants with the opportunities to connect with their family members, loved ones, and friends.
2. Connection to one’s community and neighborhood (e.g., “*Parks are about the future of our communities and neighborhoods. I feel like by participating in this study I’m making a difference for my city, I’m shaping the future of my city*”)	Urban green space makes people feel connected to their local communities and broader physical neighborhood.
3. Connection to self (e.g., “*Parks are great places for relaxation; place where you can leave all the problems behind, connect with yourself, immerse yourself in complete rest and relaxation*”)	Parks constitute a space for reflective self-exploration that facilitate personal growth and enhanced self-concept.
4. Connection to nature (e.g., “*I think it is very important that more people go to parks to be close to nature—I see in the summer people bringing blankets, having small picnics, and enjoying the greenery around them*”)	Many participants consider connection to nature as an important driving force for visiting their local parks.
